# Identification of a novel conserved mixed-isoform B56 regulatory subunit and spatiotemporal regulation of protein phosphatase 2A during *Xenopus laevis *development

**DOI:** 10.1186/1471-213X-7-139

**Published:** 2007-12-19

**Authors:** Sungmin Baek, Joni M Seeling

**Affiliations:** 1 Department of Biology, City University of New York, Queens College, 65-30 Kissena Blvd., Flushing, NY 11367, USA

## Abstract

**Background:**

Wnt signaling is a key regulator of development and tumorigenesis. Protein phosphatase 2A (PP2A), which consists of a catalytic C, a structural A, and a regulatory B subunit, plays diverse roles in Wnt signaling through its B56 subunits. B56 is a multigene family encoding for proteins with a conserved core domain and divergent amino- and carboxy-termini. Ectopic B56α and B56γ reduce β-catenin abundance and B56α reduces Wnt-dependent transcription, suggesting that B56α and B56γ inhibit Wnt signaling. In contrast, B56ε is required for Wnt signaling. Knowledge of where and when B56 subunits are expressed during *Xenopus *development will aid in our understanding of their roles in Wnt signaling.

**Results:**

We have undertaken expression analyses of B56α and B56γ in *Xenopus laevis*. We cloned *Xenopus *B56α; it is 88% identical to human B56α. *Xenopus *B56γ is 94% identical with human B56γ, however, a novel evolutionarily conserved mixed-isoform transcript was identified that contains a B56δ-like amino-terminal domain and a B56γ core domain. The B56δ-like variable domain exon is located upstream of the B56γ variable domain exon at the human B56γ locus, suggesting that the mixed-isoform transcript is due to alternative splicing. B56γ transcripts with different 3' ends were identified that lack or possess a 35 base pair sequence, resulting in either a transcript similar to human B56γ1, or an uncharacterized evolutionarily conserved sequence. Real time RT-PCR analyses revealed that B56α is expressed at moderate levels before the midblastula transition (MBT), at reduced levels during gastrulation and neurulation, and at high levels during organogenesis, while B56γ is expressed at low levels until organogenesis. B56α is enriched in the ventral hemisphere pre-MBT, while B56γ is ventrally enriched post-MBT. Aα, Aβ, Cα and Cβ are expressed in early *Xenopus *development, suggesting the presence of a functional heterotrimer.

**Conclusion:**

Our data suggest that B56 functional diversity is achieved in part through the synthesis of a novel mixed-isoform B56δ/γ transcript. Our data also suggest that B56α functions pre-MBT, inhibiting Wnt signaling on the ventral side of the embryo, and again during organogenesis, while B56γ functions primarily post-MBT.

## Background

Wnt ligands can activate canonical or planar cell polarity Wnt pathways. In canonical signaling, Wnt activates β-catenin-dependent transcription via a phosphorylation-regulated signal transduction cascade. In the absence of Wnt, casein kinase Iα (CKIα) primes β-catenin by phosphorylating Ser45, which is required for glycogen synthase kinase 3β (GSK3β) to phosphorylate three upstream Ser/Thr residues [1,2]. GSK3β also phosphorylates adenomatous polyposis coli (APC) and axin [3]. GSK3β, β-catenin, APC, and axin comprise the core components of the β-catenin degradation complex. APC and axin promote the interaction of GSK3β with β-catenin, and therefore also promote its phosphorylation [4]. Phosphorylated β-catenin is ubiquitinated and degraded by the proteasome. When Wnt binds to LRP5/6 and frizzled (fz) coreceptors, an intracellular signaling cascade is activated that includes axin binding to LRP5/6, and disheveled (dsh/dvl) phosphorylation and binding to fz [5-7]. These events lead to destabilization of the β-catenin degradation complex, reduced β-catenin phosphorylation, and increased β-catenin abundance. β-catenin then forms a complex with a Lef/Tcf transcription factor, activating transcription of dorsalizing factors in early *Xenopus *development and cell cycle regulators in mammalian cells [8-10].

Although phosphorylation cascades have been intensely studied, relatively little is known about the role that phosphatases play in them. Phosphatase catalytic subunits are bound to targeting and regulatory subunits in multimeric complexes. PP2A is an abundantly expressed Ser/Thr phosphatase that has roles in DNA replication, cell cycle control, apoptosis, development, and tumorigenesis [11]. The inhibition of PP2A can cause cell proliferation and aberrant development. The phosphatase inhibitor okadaic acid induces skin and gastrointestinal tract cancers, and four mutations in a PP2A subunit, Aα, identified in human tumors, have reduced PP2A function [12-14].

PP2A is comprised of a structural A, catalytic C, and regulatory B subunit. The B subunit confers substrate specificity and subcellular localization on the PP2A holoenzyme [15,16]. There are three distinct families of B subunits: B55, B56, and PR72. There may be as many as sixty distinct PP2A holoenzymes, since the A and C subunits, as well as each B subunit family, are encoded by multiple genes. B56 is the largest family of B regulatory subunits [15,17-20]. In humans, B56 is encoded by five highly related and widely expressed genes (B56α, B56β, B56γ, B56δ, and B56ε) that share a 71–88% identical 400 amino acid central core domain and highly variable amino- and carboxy-terminal extensions. The B56 family consists of two subfamilies based on amino acid similarity, B56α/β/ε and B56δ/γ [15]. The three-dimensional structure of a PP2A heterotrimer containing human B56γ1 has recently been determined [21]. B56γ1 forms eight pseudo-HEAT repeats (named for the four founding proteins: huntington, elongation factor 3, PP2A A, and TOR1), each of which is comprised of two antiparallel α-helices connected by intra-repeat loops. B56γ1 binds to the A subunit through the "basal" surface of pseudo-HEAT repeats 2, 4, and 6, and to the C subunit through its intra-repeat loops. The B56 subunit is proposed to bring about isoform specificity both through its amino- and carboxy-termini and through the "apical" surface of its pseudo-HEAT repeats [21].

B56α/β/ε subfamily members have roles in both canonical and planar cell polarity Wnt signaling. In many cases, B56 is inhibitory to canonical Wnt signaling. B56α reduces β-catenin abundance and Wnt-dependent gene expression in multiple experimental systems [22]. B56α's ability to reduce β-catenin abundance depends on the phosphatase activity of PP2A, since the inhibition of this activity in mammalian cells results in increased β-catenin abundance. Importantly, PP2A B56α, A, and C subunits each reduce Xwnt-8-induced secondary axes, suggesting that B56α increases B56α-specific PP2A activity rather than reducing nonB56α activity by sequestration of the PP2A AC heterodimer. In addition, B56α reduces the half-life of β-catenin in an *in vitro *β-catenin degradation assay, and the increased half-life of β-catenin in the absence of PP1 and PP2A catalytic subunits is restored by the addition of the C subunit of PP2A, but not PP1 [23]. Epistasis and coimmunoprecipitation data suggest that PP2A:B56α is a component of the β-catenin degradation complex, and that CKIδ/ε dissociates PP2A from the complex [23,24]. In addition, ΔNp63, an oncogenic p53 paralog, appears to activate Wnt signaling through its ability to bind to B56α in the absence of other PP2A subunits, preventing B56α from activating GSK3β [25]. Wdb, a B56α/β/ε family member most closely related to B56ε, influences both canonical and planar cell polarity Wnt signaling during *Drosophila *and zebrafish development. Wdb^IP^, a partial loss-of-function allele of wdb in *Drosophila*, causes wing and eye outgrowths in a homozygous state, indicative of excessive Wnt signaling, while morpholino antisense oligonucleotides (MOs) against two zebrafish wdb genes together induce planar cell polarity defects at a low dose and dorsalization at a high dose [26]. In contrast, *Xenopus *B56ε activates canonical Wnt signaling, functioning between Wnt and dsh/dvl to positively regulate Wnt signaling in dorsal development, midbrain-hindbrain boundary formation, and neural tube closure [27].

There is growing evidence that B56γ functions as a tumor suppressor. A B56γ deletion mutant is unable to dephosphorylate paxillin, and enhances cell spreading as well as metastasis [28]. The small t DNA tumor virus antigen (sm t) enhances cell growth through inhibition of B56-specific PP2A activity [12,29-31]. In fact, the B56γ binding site of sm t is required for sm t to activate Wnt signaling and transform human papilloma virus-infected keratinocytes, an *in vivo *model of cervical cancer progression [32]. In mouse, the transgenic overexpression of B56γ in lung tissue reduces β-catenin abundance and disrupts distal lung differentiation, resulting in neonatal death [33]. To better understand the distinct roles of the B56 family of PP2A regulatory subunits in early *Xenopus *development, we cloned *Xenopus laevis *B56α, analyzed B56α and B56γ sequences, and undertook a quantitative analysis of the expression of B56α, B56γ, and B56ε using real time RT-PCR on RNA collected from *Xenopus laevis *embryos at several points in development in both whole and hemisected embryos. We found dynamic and dissimilar expression patterns, reinforcing the hypothesis that B56α, B56γ, and B56ε have distinct roles in development and in their ability to influence Wnt signaling.

## Results

**Figure 1:**
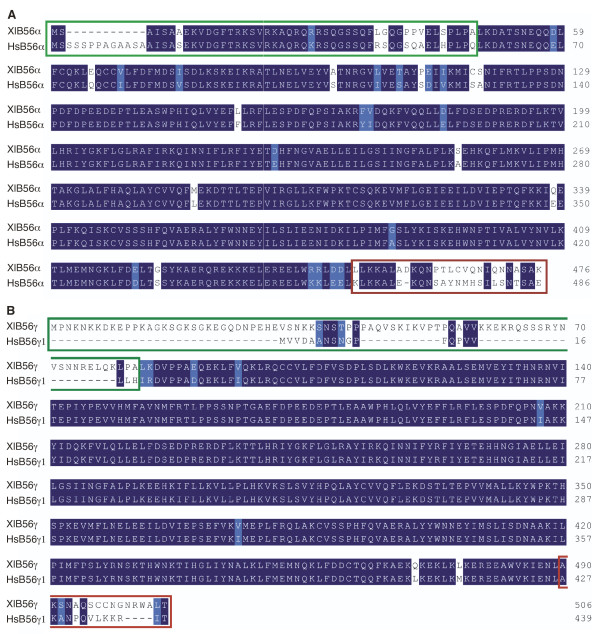
**Alignment of *Xenopus laevis *and human B56α and B56γ proteins**. Dark blue regions signify identity, light blue regions signify similarity, and dashes signify gaps. The amino-terminal variable domain is boxed in green, while the carboxyl-terminal variable domain is boxed in red. **A**. Sequences of *Xenopus laevis *(Xl) and human (Hs) B56α proteins are aligned. **B**. Sequences of *Xenopus laevis *(Xl) and human (Hs) B56γ proteins are aligned.

To facilitate our study of the role of B56 subunits during *Xenopus *development, a full-length *Xenopus *B56α cDNA was isolated from a *Xenopu*s maternal cDNA library using human B56α as a probe. B56α is highly conserved between humans and *Xenopu*s, the protein sequences are 88% identical and 92% similar overall. The proteins are more conserved in the core domain (93% identity) than in the amino- and carboxy-terminal variable domains (66% and 46% identity, respectively) (Fig. 1A). This high level of sequence conservation suggests the conservation of B56α function between frog and humans.

**Figure 2:**
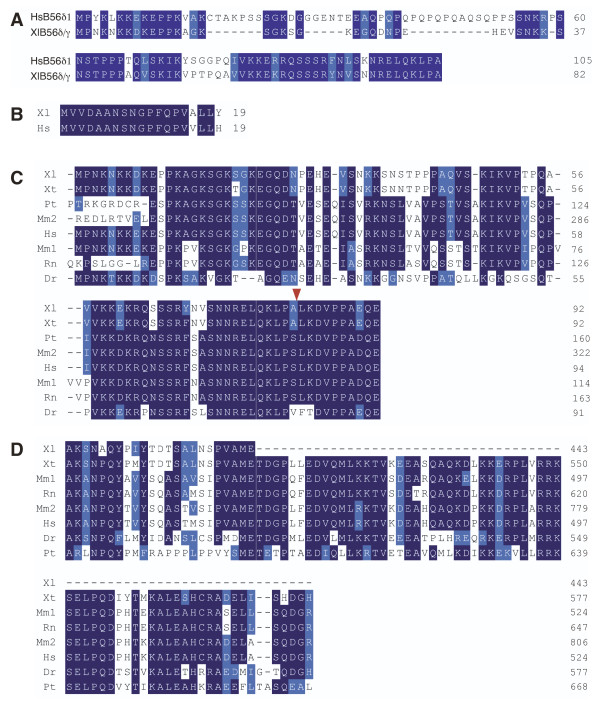
**Alignment of B56γ amino- and carboxy-terminal variable domains**. Sequence alignments are annotated as described in Figure 1. **A**. Alignment of *Xenopus laevis *B56δ/γ and human B56δ amino-terminal variable domains. **B**. Alignment of *Xenopus laevis *B56γ/γ and human B56γ amino-terminal variable domains. **C**. Alignment of *Xenopus laevis *(Xl), *Xenopus tropicalis *(Xt, GI:39645667), Pan troglodyte (Pt, GI:114654932), Macaca mulatta (Mm2, GI:109084945), human (Hs, GI:47077243), Mus musculus (Mm1, GI:37359748), Rattus norvegicus (Rn, GI:109478743), and Danio rerio (Dn, GI:113674011) B56δ/γ sequences from the B56δ-like amino-terminal variable region through the first ten amino acids of the B56γ core domain. The division between the variable domain and the core domain is marked by an arrowhead. **D**. Alignment of an alternative long form of the B56γ carboxy-terminal variable domain with the species described in C; *Xenopus tropicalis *(GI:45361341), Mus musculus (GI:71153488), Rattus norvegicus (GI:109478743), Macaca mulatta (GI:109084945), human (GI:31083259), Danio rerio (GI:113674011), Pan troglodyte (GI:114607473).

We identified a *Xenopus *B56γ cDNA from sequence databases that has 94% identity and 96% similarity to human B56γ1. The core domain is highly related to human B56γ1 (98% identical), but the *Xenopus *and human genes are divergent at their amino- and carboxy-termini (15% and 38% identity, respectively). When the amino-terminus was examined more closely, we found that it had higher identity to B56δ than to B56γ, and we termed this mixed-isoform transcript B56δ/γ. Sequence databases were then screened for additional *Xenopus laevis *B56γ sequences, and a B56γ cDNA clone was found that had a B56γ-like amino-terminus (B56γ/γ). To verify that B56δ/γ and B56γ/γ were representative of actual mRNA transcripts and were not cloning artifacts, RT-PCR was carried out with RNA from stage 32 embryos with upstream primers specific for either the *Xenopus laevis *B56δ or B56γ 5' variable domain, and a downstream primer specific to the B56γ core domain. The sequencing data obtained with the products from each of these reactions concur with our initial findings and suggest the presence of two B56γ transcripts in *Xenopus laevis *with alternative 5' ends. The B56δ/γ mixed-isoform transcript encodes an 82 amino acid amino-terminal domain that is 66% identical to human B56δ, while the B56γ/γ transcript encodes a 19 amino acid domain that is 89% identical to human B56γ (Fig. 2AB). In both cases, the core domain is identical, and has 98% identity to human B56γ, compared to 89% identity to the human B56δ core domain. (Fig. 1B and data not shown). A search of sequence databases identified seven species that possess this novel mixed-isoform B56δ/γ transcript: *Xenopus tropicalis*, humans, chimpanzee, rhesus monkey, mouse, rat, and zebrafish (Fig. 2C). Therefore, this novel B56δ/γ transcript is evolutionarily conserved.

**Figure 3:**
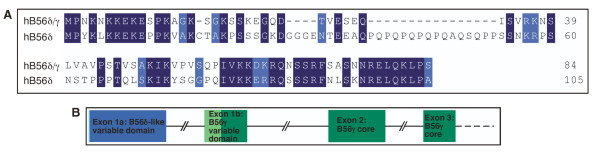
**The B56δ-like amino-terminal variable domain is distinct from B56δ and encoded at the B56γ locus**. **A**. Human B56δ and human B56δ-like amino-terminal variable domains are 51% identical. Sequence alignments are annotated as described in Figure 1. **B**. The exon encoding the B56δ-like amino-terminal variable domain (Exon 1a, light blue and dark blue bars represent noncoding and coding sequences, respectively) is upstream of the exon encoding the B56γ amino-terminal variable domain (Exon 1b, light green and dark green bars represent noncoding and coding sequences, respectively) in the human B56γ gene (chromosome 14p32.2). The introns are represented by lines and are shown in scale with one another, but not in scale with the exons.

To further study the origin of the mixed-isoform B56δ/γ transcript, we examined the human genome database. The human B56δ 5' variable domain upstream of the B56γ core is distinct from the human B56δ 5' variable domain upstream of the B56δ core, being 51% identical, and therefore is termed B56δ-like (Fig. 3A). In fact, the human B56δ and B56δ-like sequences are more highly related to the *Xenopus laevis *sequence than each other, suggesting that the B56δ and B56γ genes are the result of a duplication of the B56δ/γ gene. If the novel mixed-isoform B56δ/γ transcript is the result of alternative splicing of the B56γ gene, then the B56δ-like 5' variable domain should be present on chromosome 14p32.2, where B56γ is located [34]. Indeed, the B56δ-like 5' variable domain is present on chromosome 14p32.2 in an exon upstream of the B56γ 5' variable domain exon (Fig. 3B). The B56δ/γ mixed-isoform transcript is thus a splice-variant from the B56γ locus.

Our data, and that of others, has shown that *Xenopus laevis *possesses B56α, B56γ (both B56δ/γ and B56γ/γ), and B56ε orthologs. In addition, we found a partial cDNA of B56β (XDB3.2 clone number rXL259o17ex) but we were unable to find evidence of expression of a B56δ core domain. Since the sequencing of the *Xenopus tropicalis *genome is near completion, and because of the high level of relatedness between *Xenopus tropicalis *and *Xenopus laevis*, we searched *Xenopus tropicalis *sequences to determine its complement of B56 genes, with the expectation that this is likely to also represent the B56 complement in *Xenopus laevis*. We used both *Xenopus laevis *and human B56 isoform sequences to screen sequence databases for *Xenopus tropicalis *B56 genes. We found B56α, B56γ (both B56δ/γ and B56γ/γ), B56ε, and B56β orthologs, but no isoform with a B56δ core domain. B56β is expressed most highly in adult brains [17,35], and the *tropicalis *B56β was sequenced from an adult brain cDNA library. Although we did not find a *laevis *or *tropicalis *B56δ core domain, *Danio rerio *does possess a B56δ sequence containing the B56δ core domain (GI:40353001), suggesting either that *Xenopus *has a B56δ core domain but it has not yet been sequenced, or that *Xenopus *has lost this gene.

Alternative splicing occurs at the 3' end of human B56γ, resulting in B56γ1, B56γ2, and B56γ3 transcripts [34]. We found two alternative forms for the 3' end of the *Xenopus laevis *B56γ transcript. One isoform is most similar to human B56γ1 (Fig. 1B). The second form has an insertion of thirty-five nucleotides downstream of the core domain that leads to a frame-shift. The resulting open reading frame does not have significant identity to the 3' end of human B56δ, B56γ1, B56γ2, or B56γ3. However, this sequence is conserved in *Xenopus tropicalis*, humans, chimpanzee, rhesus monkey, bovine, dog, bovine, rabbit, mouse, rat, chicken, puffer fish, and zebrafish (Fig. 2D and data not shown), and is expressed in mouse testis [36]. Therefore, this alternative 3' end of the B56γ transcript is also evolutionarily conserved.

Knowledge of where and when B56α and B56γ are expressed during *Xenopus *development will aid in our understanding of their role in Wnt signaling. To determine the expression pattern of B56α and B56γ, real-time reverse transcriptase-polymerase chain reaction (RT-PCR) was carried out on *Xenopus *embryos at several stages in early development. The expression patterns of B56α and B56γ were compared to that of B56ε, whose expression pattern has previously been reported, so that even slight differences in their expression patterns could be detected [27].

**Figure 4:**
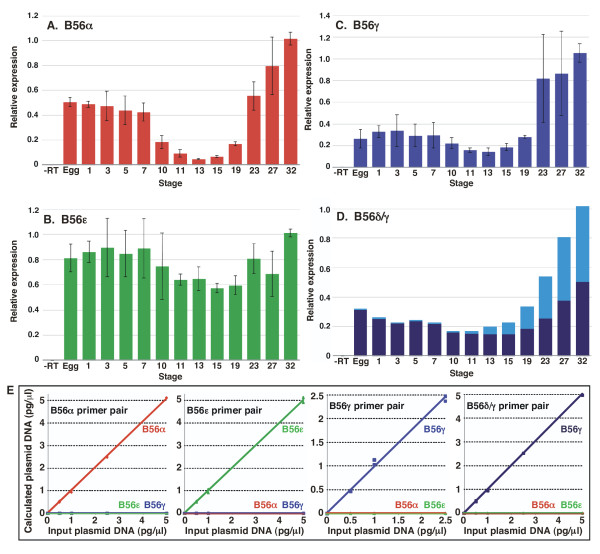
**B56α and B56γ are differentially expressed during early *Xenopus *development**. RNA was purified from *Xenopus laevis *embryos at the indicated stages, and real time RT-PCR was carried out. A standard curve was done from stage 32 embryos, and signals were normalized to that stage. **A**. B56α is expressed at approximately 45% of the stage 32 value prior to MBT, at approximately 10% of the stage 32 value during gastrula and neurula stages, and its expression gradually increases to its highest expression level during organogenesis. **B**. The expression level of B56ε is relatively unchanged from the egg to stage 32. **C**. The expression level of B56γ is highest during organogenesis and is approximately 25% of that value during earlier stages (unfertilized egg to stage 19). **D**. The mixed-isoform B56δ/γ transcript is expressed at moderate levels until organogenesis (dark blue bars), while B56γ/γ is not significantly expressed until neurulation (light blue bars); both B56δ/γ and B56γ/γ display increased expression during organogenesis. **E**. A dose response curve of real time RT-PCR amplification data shows that B56α, B56γ, and B56ε primer pairs are isoform specific. Input plasmid DNA concentrations are plotted against their concentration calculated from the amplification of the correct primer pair/template combination.

Because B56 genes are members of a highly conserved family, we first verified that the primer pairs that we designed were isoform specific. We designed B56α, B56γ, or B56ε primer pairs to amplify their 3' variable regions. We also designed B56γ primer pairs to amplify either the B56δ-like or B56γ 5' variable regions. The B56γ primer pair at the 3' end of the gene amplified all of the B56γ transcripts identified here: transcripts with either the B56δ-like or B56γ 5' variable regions, as well as both the short and long 3' alternative splicing products. Real-time PCR was then performed with each B56 primer pair and B56α, B56γ, or B56ε plasmid templates. Each primer pair specifically amplified only the corresponding isoform (Fig. 4E). These isoform-specific primer pairs were then used to examine the expression pattern of B56α, B56γ, and B56ε in early *Xenopus *development. B56α is expressed at approximately 45% of the stage 32 value prior to MBT (egg – stage 7), at approximately 10% of the stage 32 value during gastrula and neurula stages (stages 10 – 19), and its expression gradually increased to its highest expression level during organogenesis (stages 23–32) (Fig. 4A). The expression level of all of the B56γ transcripts described here was highest at stage 32 (Fig. 4C). B56γ expression was approximately 25% of the stage 32 value from the egg to neurula stages (egg to stage 19), and increased during organogenesis. The B56δ/γ transcript was expressed at moderate levels until organogenesis, while B56γ/γ was not significantly expressed until neurulation, while B56δ/γ and B56γ/γ both displayed increased expression during organogenesis (Fig. 4D). The expression level of B56ε was relatively unchanged from the egg to stage 32 (Fig. 4B). This data suggests that B56α has at least two functions during early *Xenopus *development, one during cleavage stages and another during organogenesis. In contrast, B56γ/γ appears to have its primary function during organogenesis, while B56δ/γ may function throughout early development.

**Figure 5:**
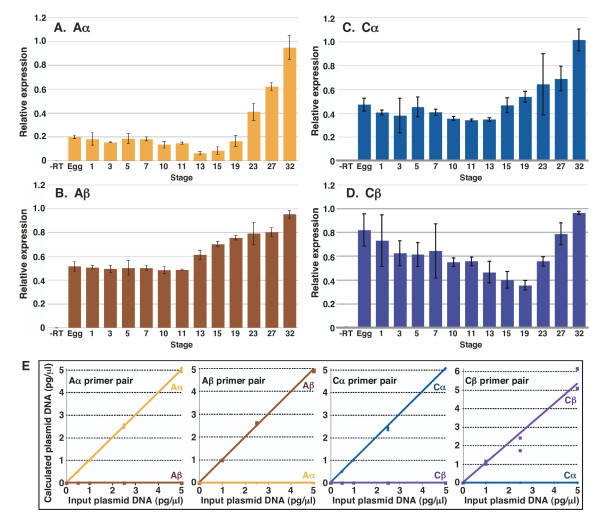
**PP2A Aα, Aβ, Cα, and Cβ are differentially expressed during early *Xenopus *development**. RNA was purified from *Xenopus laevis *embryos at the indicated stages, and real time RT-PCR was carried out. A standard curve was done from stage 32 embryos, and signals were normalized to that stage. **A**. Aα is expressed at low levels until organogenesis. Aβ **(B) **and Cα **(C) **are expressed at moderate levels through gastrulation, and at progressively increasing levels during neurulation and organogenesis. **D**. Cβ is expressed at similar levels from the egg to stage 32, with the exception of its reduced expression during neurulation. **E**. A dose response curve of real time RT-PCR amplification data shows that Aα, Aβ, Cα, and Cβ primer pairs are isoform specific. Input plasmid DNA concentrations are plotted against their concentration calculated from the amplification of the correct primer pair/template combination.

If B56 subunits function as regulatory subunits of PP2A heterotrimers during *Xenopus *development as we hypothesize, then the A and C subunits should be concurrently expressed. Vertebrates possess two A isoforms, Aα and Aβ, as well as two C isoforms, Cα and Cβ. We carried out real time RT-PCR to examine the expression patterns of Aα, Aβ, Cα, and Cβ during early *Xenopus laevis *development. Because the A and C isoforms are highly conserved (the coding regions of Aα and Aβ are 73% identical, and Cα and Cβ are 79% identical), we first performed real-time PCR with Aα, Aβ, Cα, and Cβ primer pairs and Aα, Aβ, Cα, or Cβ plasmid templates to verify the isoform specificity of our primer pairs. Each primer pair specifically amplified only the corresponding isoform (Fig. 5E). These isoform-specific primer pairs were then used to examine the expression pattern of Aα, Aβ, Cα, and Cβ in early *Xenopus *development. Aα was expressed at low levels until organogenesis, whereas Aβ and Cα were expressed at moderate levels from the egg through gastrulation, and at progressively increasing levels during neurulation and organogenesis (Fig. 5A–C). Cβ was expressed at similar levels from the egg to stage 32, with the exception of its reduced expression during neurulation (Fig. 5D). Therefore, each of the components of the PP2A heterotrimer is present during early *Xenopus *development, suggesting that competent PP2A heterotrimers containing B56α, B56γ, and B56ε are present during early *Xenopus *development.

**Figure 6:**
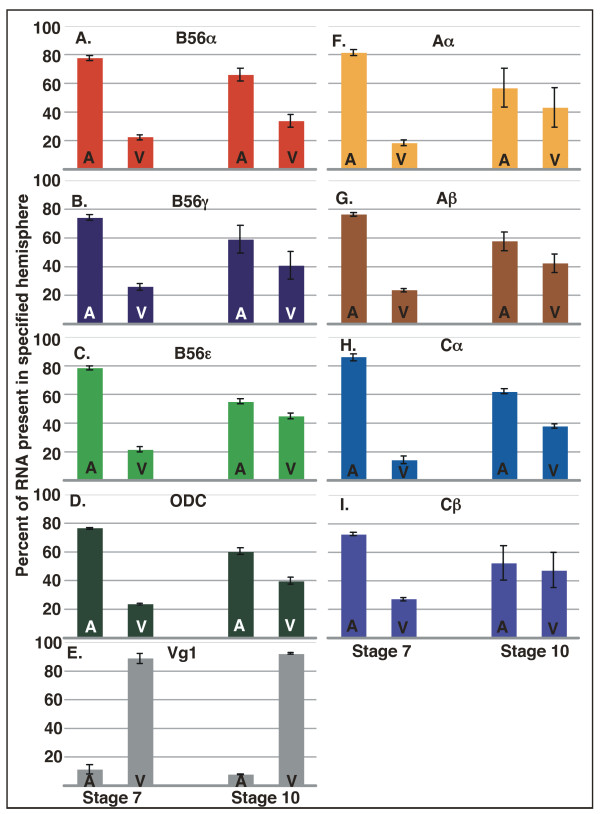
**PP2A subunits are enriched in the animal hemisphere of *Xenopus *embryos**. *Xenopus laevis *embryos at stages 7 and 10 were hemisected into animal and vegetal halves. RNA was purified from the hemisected halves and real time RT-PCR was carried out. The data is presented as the percentage of the total signal that is present in the specified hemisphere ± SD. B56α (A), B56γ (B), B56ε (C), Aα (F), Aβ (G), Cα (H), and Cβ (I) are enriched in the animal hemisphere of stage 7 and 10 *Xenopus *embryos. Dissection control ODC (D) is enriched animally, while Vg1 (E) is enriched vegetally. A = animal, V = vegetal.

To further explore the expression pattern of PP2A subunits during *Xenopus *development, we localized their expression by real-time RT-PCR analyses of hemisected embryos. In general, RNA is more abundant in the animal hemisphere of the early *Xenopus *embryo because yolk plasm in the vegetal hemisphere reduces cytoplasmic volume. Total RNA, as well as poly(A)+ RNA, is two to four fold more abundant animally versus vegetally [37,38]. This differential distribution of RNA continues into gastrula stage embryos [37]. However, a number of maternal RNAs are differentially localized beyond, or contrary to, this inclination on the animal/vegetal axis. RNAs that will become germ plasm constituents or RNAs required for proper cell fate decisions, such as Vg1, are enriched vegetally [39]. Other RNAs, such as Tcf-1 and β-TrCP, are enriched animally [39]. We analyzed the expression of PP2A subunits in animal/vegetal hemisected embryos. B56α, B56γ, B56ε, Aα, Aβ, Cα, and Cβ were animally enriched approximately 4-fold at stage 7 and 1.5-fold at stage 10 (Fig. 6A–D, F–I). Ornithine decarboxylase (ODC), a housekeeping gene, had a similar distribution, whereas Vg1, a dissection marker, was vegetally enriched approximately nine-fold at each of these stages (Fig. 6D,E). Therefore, the PP2A subunits that we examined, like most maternal RNAs, are enriched in the animal hemisphere.

**Figure 7:**
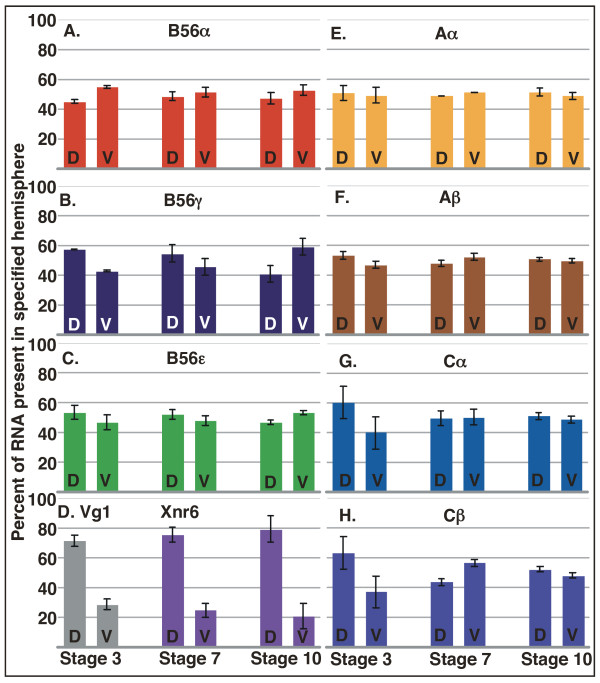
**The dorsoventral distribution of PP2A subunits in *Xenopus *embryos**. *Xenopus laevis *embryos at stages 3, 7, and 10 were hemisected into dorsal and ventral halves. RNA was purified from the hemisected halves and real time RT-PCR was carried out. The data were normalized to ODC expression levels, and are presented as the percentage of the total signal present in the specified hemisphere. PP2A A and C subunits Aα (E), Aβ (F), Cα (G), and Cβ (H) are relatively equally distributed dorsoventrally or slightly enriched dorsally. However, B56α (A) at stage 3 and B56γ (B) at stage 10 are more abundant ventrally. Dissection controls Vg1 (stage 3) and Xnr6 (stages 7 and 10) are enriched dorsally (D). D = dorsal, V = ventral.

RNA is not equally distributed dorsoventrally in *Xenopus *embryos. Total RNA is much higher in the dorsalmost region of the embryo at stage 3, while poly(A)+ RNA is equally distributed in the ventral and dorsal halves, causing a higher poly(A)+ to total RNA ratio on the ventral side of the embryo [37]. Several RNAs display differential dorsal/ventral localization during early *Xenopus *development [40]. In stage 5 embryos, for example, EF1α, goosecoid, and noggin are enriched on the dorsal side of the embryo, while Wnt8b is ventrally enriched, where it is required to inhibit dorsal cell fate [40]. Since B56 subunits influence dorsoventral body axis formation, we determined if there are any dorsoventral differences in their expression [23,27,33]. We carried out real-time RT-PCR on ventral/dorsal hemisected embryos at stages 3, 7, and 10 to determine if B56α or B56γ are differentially localized during dorsoventral axis specification, as well as to localize Aα/β and Cα/β expression. We found that most of the examined PP2A subunits were relatively equally distributed dorsoventrally, or were slightly higher dorsally. However, B56α at stage 3 and B56γ at stage 10 are an exception to this pattern, since they are more abundant ventrally (Fig. 7). Dissection markers Vg1 (stage 3) and Xnr6 (stages 7 and 10) were 2.5 – 4 fold higher dorsally, as expected (Fig. 7D, [41,42]). These expression patterns suggest that B56α may be inhibiting Wnt activity on the ventral side of the embryo in the initial stages of dorsal axis specification, whereas B56γ may carry out this function post-MBT. However, the magnitude of the differential localizations of B56α and B56γ is not large, and posttranscriptional mechanisms may also play a role in the spatial regulation of PP2A:B56 heterotrimers in the early embryo.

## Discussion

Humans possess five B56 family members: B56α, B56β, B56γ, B56δ, and B56ε. We isolated the *Xenopus laevis *B56α gene and characterized B56γ transcripts present during early *Xenopus laevis *development. We also found a partial B56β cDNA; B56ε has previously been isolated [43]. The B56δ core domain has not been found in *Xenopus laevis *cDNA library sequences from public databases, suggesting that it is not present in early stage embryos. The B56δ core domain is highly expressed in the brain [35,44], and is present in *Danio rerio*, but has not yet been sequenced in *laevis *or *tropicalis*, suggesting that the appropriate adult library may not have been sequenced, or that *Xenopus *has lost the B56δ gene.

B56 proteins are comprised of a conserved central core domain and divergent amino- and carboxy-terminal extensions. The core interacts with the highly conserved A and C subunits, while the variable domains provide substrate specificity by binding diverse substrates. B56α and B56γ core domains are highly conserved between humans and *Xenopus*, while the amino- and carboxy-terminal extensions are less well conserved. In fact, each residue in human B56α and B56γ that interacts directly with the A and C subunits [21] is identical in *Xenopus laevis*. The relative conservation of the core domains and divergence of the amino- and carboxy-terminal variable domains of B56α and B56γ between humans and *Xenopus *is likely to reflect their interactions with disparate types of protein binding partners: the core interacting with A and C subunits, and the amino- and carboxy-terminal domains interacting with substrates, proteins that are not likely to be as highly conserved as the A and C subunits.

Our data suggest that diversity of B56 function is achieved in part through the synthesis of alternative transcripts that result in distinct amino- and carboxy-terminal domains. We identified a novel mixed-isoform B56δ/γ transcript in *Xenopus laevis *comprised of a B56δ-like amino-terminal variable domain and a B56γ core domain, as well as a B56γ transcript possessing an alternative 3' end. Each of these variants is evolutionarily conserved, and is present in humans. The intense conservation of these novel B56γ transcripts suggest that they are functional, because if they were not functional, their sequence would rapidly drift away from the consensus. The identification of a mixed-isoform B56δ/γ transcript suggests that alternative splicing leads to a "fine-tuning" of PP2A substrate specificity.

The expression of several B subunits during *Xenopus *development has been examined. Northern blotting analyses show that the B55 subunit is present at constant levels from the egg to metamorphosis, and present in the adult in the ovary, testis, muscle, and liver [45]. The expression of multiple PR72 isoforms was examined in *Xenopus *oocytes and adult tissues by hybridization detection of RT-PCR products [46]. XPR130 is expressed at low levels in the oocyte and at higher levels in the liver, gall bladder, spleen, heart, and muscle, while XPR70 is expressed at high levels in oocytes and each of the tissues mentioned above. B56ε, which is required for Wnt signaling, is expressed maternally and throughout early *Xenopus *development [27]. Its expression is uniform in the embryo until MBT, when its expression, as determined by *in situ *hybridization, becomes localized to the dorsal marginal zone in early gastrulation and expands to the entire marginal zone during midgastrulation. B56ε is expressed in the neural ectoderm during neurulation and in the eyes and branchial arches in the tadpole. We demonstrate here that B56α and B56γ, both of which have Wnt-inhibitory activities, display dynamic expression patterns in early *Xenopus *development. B56α is expressed at moderate levels before MBT, when it may inhibit Wnt pathway activity on the ventral side of the embryo, at reduced levels during gastrulation and neurulation, and at high levels during organogenesis, while B56γ is expressed at moderate levels until organogenesis. B56γ has been shown to have a crucial role during lung development in the mouse, which is consistent with our finding that it is highly expressed during organogenesis [33].

Prior to this report, Aβ and Cα were known to be expressed in *Xenopus *embryos, however, we detected Aα, Aβ, Cα, and Cβ expression during early *Xenopus *development. PP2A A and C subunit expression has previously been examined by Northern and western blotting. PP2A Cα and Cβ were both undetectable until stage 35 by Northern blotting, when Cα is expressed at higher levels than Cβ; in adults, they are most abundant in the ovary and heart [47]. However, a more recent report using Northern blotting shows that Cα is expressed at similar levels from the egg through the gastrula stage [48]. Our results concur with these findings, but show that both Cα and Cβ RNAs are present in the egg and throughout early development. Cα and Cβ expression increases during organogenesis, when Van Hoof et al. first detected expression ([47], Fig. 5C–D). The expression of PP2A Aα and Aβ isoforms in *Xenopus *has previously been studied by western blotting, where it was found that Aβ is expressed in the oocyte and both Aα and Aβ are expressed in skeletal muscle [49], and by Northern blotting, where it was found that Aβ is expressed at similar levels from the oocyte to metamorphosis, whereas Aα expression is undetectable until stage 35 [45]. In adults, Aβ is highly expressed in the ovary, but expressed at reduced levels in all other tissues examined, while Aα is highly expressed in muscle, heart, and testes, and moderately expressed in liver, lung, and spleen [45]. Our results concur with these findings, but presumably due to the increased sensitivity of real-time RT-PCR as compared to Northern blotting, Aα was found to be expressed at low levels prior to organogenesis, after which its expression sharply increased, while expression was relatively more constant for Aβ (Fig. 5A–B). Since PP2A functions as a heterotrimer, the presence of Aα/β and Cα/β isoforms in early *Xenopus *development suggests that B56α and B56γ, as well as any other B subunits expressed during this time, will associate with A and C subunits, forming a functional heterotrimer.

The enrichment of B56α RNA on the ventral side of a 4 cell-stage embryo, against the trend of the A and C subunits to be enriched dorsally or equally distributed, suggests that B56α's ventral enrichment is significant. This, along with our previous data showing that B56α can inhibit Wnt signaling [22,23], suggest that B56α is functioning pre-MBT in ventral cells to inhibit dorsal cell fate. Other pre-MBT dorsoventral asymmetries exemplified by ventral enrichment have been identified. Wnt8b is enriched ventrally at the 16 cell-stage, where it represses dorsal cell fate [40]. Dorsal-specific signaling does occur pre-MBT, since Wnt-dependent transcription of Xnr5 and Xnr6 is detected as early as the 256 cell-stage [42]. The enrichment of B56γ RNA on the ventral side of the embryo at stage 10, again against the trend of the A and C subunits to be enriched dorsally or equally distributed, suggests that this enrichment is significant. B56γ can reduce β-catenin abundance [33], therefore its ventral enrichment suggests that B56γ functions post-MBT to inhibit Wnt signaling. Further tests will be required to determine if there is a requirement for ventral enrichment of B56α and B56γ in specifying dorsoventral cell fate, such as antisense morpholino oligonucleotide microinjections, which are currently underway.

Distinct PP2A heterotrimers may act on different substrates, or at multiple sites within a given substrate, with opposing consequences. Wnt signaling is regulated by phosphorylation at multiple points. Numerous kinases influence Wnt signaling, and each phosphorylation step carried out by a kinase is likely to have a counterregulatory dephosphorylation step carried out by a phosphatase. Consequently, there are likely to be as many phosphatases acting in the pathway as kinases. In contrast to the plethora of kinases, there are a limited number of phosphatase catalytic subunits. Diversity of phosphatase function is achieved through variation in the bound regulatory subunit. Since PP2A is one of the most abundant phosphatases, it is reasonable to expect that PP2A has multiple roles in the pathway. Evidence from our lab and others suggest that this is the case even within the B56 family [23,27,33,43,50].

The PR72 family plays an important role in Wnt signaling. PR72 inhibits Wnt signaling through its interaction with Nkd, another Wnt inhibitor, and promotes dsh degradation [51]. Another PR72 family member, PR130, also binds to Nkd [52]. The binding of PR130 and PR72 to Nkd may be mutually exclusive, with PR72 inhibiting Wnt signaling when bound to Nkd due to its promotion of dsh degradation, and PR130 activating Wnt signaling when bound to Nkd by preventing Nkd-mediated Wnt inhibition. There are other gene families important to Wnt signaling in which different members of the gene family affect the pathway differently. For instance, CKIα inhibits the pathway by promoting β-catenin degradation through the phosphorylation of β-catenin's Ser45, while CKIδ/ε activates the pathway, possibly by destabilizing the β-catenin degradation complex [1,2,24]. Therefore, the regulation of Wnt signaling is clearly complex and cannot be completely understood until the roles of multigene families that influence it have been characterized.

The expression of B56α and B56γ in early *Xenopus laevis *development suggests that their regulation of PP2A activity at this time may influence Wnt signaling. It is of interest to note that PP2A A subunit mutations have been identified in human melanomas and colon, breast, and lung tumors [13,53-57]. Interestingly, two PP2A Aα mutant proteins initially identified in human cancers bind every PP2A subunit at near wild-type levels except B56 [13,14]. This suggests that the loss of B56-specific PP2A activity is involved in tumorigenesis, and since B56α and B56γ can inhibit Wnt signaling, this suggests that the regulation of Wnt signaling by PP2A may be a critical factor in tumorigenesis. Future studies include the localization of B56α and B56γ in early *Xenopus *development through *in situ *hybridization, which will enhance our knowledge of the spatiotemporal expression patterns of B56α and B56γ, and B56α and B56γ loss-of-function assays, each of which will help us understand in more detail how B56α and B56γ influence Wnt signaling.

## Conclusion

The activity of PP2A is regulated by the bound B subunit. The B56 family of B subunits has five genes in mammals, and our data suggest that diversity of B56 function is obtained in part through alternative splicing of the 5' and 3' variable domains. We found that the B56γ transcript can be comprised of either a B56δ-like or B56γ 5' variable domain, and either a B56γ1-like or a novel conserved sequence for its 3' variable domain. Our data also suggest that B56α functions preMBT, inhibiting Wnt signaling on the ventral side of the embryo, and again during organogenesis, while B56γ functions primarily during organogenesis.

## Methods

### Library screening and plasmids

*Xenopus laevis *maternal cDNA library in pBluescript SK+ (A. Zorn) was screened with digoxygenin-labeled human B56α synthesized by PCR, and detected with DIG Luminescent Detection Kit (Roche, Indianapolis, IN). Positive clones were sequenced by primer walking (Macrogen, Seoul, Korea). Full length *Xenopus laevis *B56γ and B56ε genes cloned in pSPORT6 were obtained from the *Xenopus *Gene Collection in the I.M.A.G.E. consortium through American Type Culture Collection (ATCC); GenBank accession number of B56α: EU307104, B56γ: BC081028, B56ε: BC084241.

### RT-PCR and sequence analysis of B56γ transcripts

RT-PCR was performed with cDNA from stage 32 embryos with the primers XlB56γ2U 5'-CCT CTT TTC CGG CAG CTA G-3' and XlB56γ2D 5'-TTC CAT TGC AAC AGG ACT-3'. PCR products were sequenced (Macrogen, Seoul, Korea). Sequence analysis was performed with ClustalW [62] and The Sequence Manipulation Suite [63].

### Real-time RT-PCR

To verify the isoform specificity of the B56α, B56γ, B56ε, Aα, Aβ, Cα, and Cβ primer pairs, real-time PCR was performed using plasmid templates. Plasmid concentrations of each family member were used (0.5 to 5 pg/μl, except B56γ (3'end): 0.5 – 2.5 pg/μl) that exceed the signals obtained with the cDNA samples as determined by their Cp values. A 20 μl reaction included 1.8 to 3 μM primers, 10 μl of 2× SYBR master mix, 2 μl of template, and 0.5 units of Uracil-DNA glycosylase (New England Biolabs, Beverly, MA). Real-time RT-PCR was done using QuantiTech SYBR green PCR kit (Qiagen, Valencia, CA) in the LightCycler 2.0 (Roche, Indianapolis, IN). The reaction program consisted of four steps: pre-incubation, quantitation, melting curve analysis, and cooling. Data analysis was performed by LightCycler 4.0 software (Roche, Indianapolis, IN). The data were plotted using the standard curve obtained using the correct DNA/primer pair combination.

Embryos were collected at different stages of development [58]. Total RNA was extracted from embryos using Trizol solution and a chloroform, isopropanol-tRNA solution. RNA was washed with 70% ethanol and resuspended in 125 μl of DEPC-treated water per 10 embryos. cDNA synthesis was done using 1 μg of total RNA with SuperScript III First-Strand Synthesis SuperMix and random hexamers (Invitrogen, Carlsbad, CA) following manufacturer's instructions. For stage-specific expression, a dilution series of stage 32 cDNA was used to generate a standard curve to determine relative cDNA concentrations and signals were normalized to stage 32; error bars represent +/- one standard deviation.

The following primers were used: XlB56α (U: 5'-TCA TAC AAA GCA GAG CGA-3', D: 5'-TTC TGC ACA CAG AGA GTA GG-3'), XlB56γ (U: 5'-TGC AGC TAA GAT TTT GCC-3', D: 5'-TTC CAT TGC AAC AGG ACT-3'), XlB56δ/γ (U: 5'-ACT CCA CAG GCT GTT GTT-3', D: 5'-CAG GTC ACT TAG AGG ATC AGA-3'), XlB56γ/γ (U: 5'-ATG GTG GTA GAT GCA GCT AA-3', D: 5'-CAG GTC ACT TAG AGG ATC AGA-3'), XlB56ε (U: 5'-ATT CAA ACG TCG TCC TTC-3', D: 5'-CCA TCA CTC CTA AGA CCT CTC-3'), XPR65α (U: 5'-GGG GAC CTT TAC TTC ATT G-3', D: 5'-CTC ATG AGA AAT GGC ACG-3'), XPR65β (U: 5'-GGG AAG CTT CAC CAG TCT T-3', D: 5'-TTC CTC AAG GAG TCC ACT G-3'), XC36α (U: 5'-GAG GGC CAG GTC AAG AGT-3', D: 5'-TCA TGA TTT CCT CGG AGG-3'), XC36β (U: 5'-GGA AAC CAG GCA GCT ATT-3', D: 5'-AAG GCA TCG TGT AAA GAT TG-3'), Vg1 (U: 5'-CCA TAC CCG CTG ACA GAA AT-3', D: 5'-CCT GCA GCC ACA CTC ATC TA-3') [59], Xnr6 (U: 5'-AAG ATT GGA TGG GGT CAT CA-3', D: 5'-ATC AGC ATG GAC AAG GGA CT-3') [60], ODC (U: 5'-GCC ATT GTG AAG ACT CTC TCC ATT C-3', D: 5'-TTC GGG TGA TTC CTT GCC AC-3') [61].

### Embryo dissections

Embryos were hemisected at stages 3, 7, and 10. The planes of hemisection were determined based on pigmentation differences for all animal/vegetal hemisections, as well as stage 3 dorsal/ventral hemisections. Stage 7 dorsal/ventral hemisection were done using embryos marked with Nile blue at stage 3, and stage 10 dorsal/ventral hemisections were done based on the location of the dorsal blastopore lip. Real-time RT-PCR was carried out on hemisected halves of embryos as described above using equal amounts of total RNA. Vg1 was used as a vegetal marker, as well as a dorsal marker for stage 3, while Xnr6 was used as a dorsal marker for stages 7 and 10 [41,42]. In each dissection, standard curves were generated using the dissected half with the higher expression level. For dorsoventral-specific expression, signals were normalized to ODC expression, while animal/vegetal dissection signals are presented directly.

## Authors' contributions

SB participated in experimental design, and carried out the library screening and real time RT-PCR. JMS conceived of the study, participated in experimental design, and drafted the manuscript. Both authors read and approved the final manuscript.
